# Three-Dimensional Visualization of Defects Formed during the Synthesis of Metal–Organic Frameworks: A Fluorescence Microscopy Study**

**DOI:** 10.1002/anie.201205627

**Published:** 2012-11-09

**Authors:** Rob Ameloot, Frederik Vermoortele, Johan Hofkens, Frans C De Schryver, Dirk E De Vos, Maarten B J Roeffaers

**Affiliations:** Center for Surface Chemistry and Catalysis, Katholieke Universiteit LeuvenKasteelpark Arenberg 23, 3001 Leuven (Belgium); Department of Chemistry, Katholieke Universiteit LeuvenCelestijnenlaan 200F, 3001 Leuven (Belgium)

**Keywords:** crystal growth, fluorescence, fluorescence microscopy, metal–organic frameworks, microporous materials


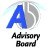


Porous crystalline frameworks are highly ordered solids in which all pores have exactly the same size and shape, as determined by the crystal structure, that is, the lattice parameters, symmetry, and coordinates of the atoms. This regular spacing of uniform nanometer-sized pores lays the foundation for the use of these materials in applications involving shape- and size-selective adsorption, catalysis, and sensing.[1–5] When the regular porous interior of such solids is disrupted by the introduction of defects, the behavior with respect to the intended application can be drastically altered.[6] Imperfections of the crystal lattice do not, however, necessarily lead to a deterioration of the material’s performance. In fact, controlled introduction of defects is standard practice for some applications of porous materials. A well-known example is ultrastable Y zeolite (USY), a key cracking catalyst used for upgrading high-boiling fractions of petroleum crude, which is prepared by partial destruction of the parent zeolite Y framework.[7] This treatment is used to control the framework composition and introduce mesoporosity in order to maximize catalyst productivity and lifetime.

Metal–organic frameworks (MOFs) are a relatively recent addition to the family of crystalline porous materials. MOFs are organic–inorganic hybrids built up from metal ion nodes linked together by organic linkers to form a three-dimensional crystal lattice.[8] Because of their extremely high surface area and porosity, the main driving force for exploring MOFs is their potential in adsorption and catalytic applications.[1–5] The effect of introducing defects in MOFs has been evaluated by numerous research groups with a background in designing and optimizing catalysts and adsorbents. The intentional and controlled introduction of defects in the MOF lattice has been mainly aimed at increasing the availability and activity of Lewis acid metal sites in the context of catalysis, typically through the incorporation of ligands lacking or bearing different functional groups.[9–11]

On the other hand, several studies where defect introduction was not intended report catalytic activity[12, 13] or adsorption strengths[14, 15] above what is expected for the intact crystal lattice. This unexpected behavior has been attributed to the presence of defect sites resulting from the synthesis or activation procedure. Although the impact of such defects on the performance of MOF materials has been reported only in a few studies, their influence is likely much more widespread; a certain level of defect sites or framework degradation is expected to be present more often than not. Variations in defect density and crystallinity account at least in part for the divergence between specific surface area measurements reported by groups applying different synthesis procedures to obtain MOF materials with the same structure.[16]

In order to clarify the relationship between the preparation conditions, defect location, and adsorptive or catalytic performance of MOFs, sufficiently sensitive tools for the detection of defect sites are needed. Several techniques have already been used to probe the degradation of MOFs. For instance, in situ X-ray diffraction (XRD)[17, 18] and scattering[19] methods have been applied to demonstrate MOF degradation and the successive formation of other phases during prolonged synthesis. While these techniques are perfectly suited to elucidate the formation process as a whole, including possible induction periods, intermediate phases etc., they are not ideal to detect the onset of defect formation. Indeed, framework degradation can be detected as a decrease in diffraction or scattering signal, but this will only be observable at an advanced stage of defect formation. Moreover, comparing different samples in more typical ex situ measurements is not trivial. Finally, as these types of measurements are typically performed on powder samples, they yield a result averaged over a multitude of crystals. Microscopic techniques, on the other hand, can reveal the location, size, and even orientation of crystal features at the single-particle level. Scanning probe microscopy has been employed to study single MOF crystals and revealed defects such as cracks along certain crystallographic directions.[20, 21] However, while giving a very detailed picture, the information resulting from scanning probe techniques is strictly limited to the outer surface, which precludes determining the presence and propagation of defects and defect planes within the crystal body.[21]

Here we demonstrate that confocal fluorescence microscopy is a powerful tool for the three-dimensional visualization of defects formed in single crystals of MOFs based on carboxylate linkers. The fluorescence signal in this study is generated by the catalytic oligomerization of the probe molecule furfuryl alcohol on acidic sites, which result, for example, from cleavage of metal–carboxylate bonds during defect formation.[22] This small fluorogenic probe has previously proven instrumental in mapping the reactivity of acidic zeolite materials with detection limits even down to the single-molecule level.[23–25] The small size of the furfuryl alcohol molecule permits diffusion and visualization of reactivity in sub-nanometer pores. The advantage of such catalytic fluorescence generation over fluorescent probe molecules that stain sites for which they have high affinity is that the signal is amplified as long as the sites to be visualized are allowed to react. In addition, and of special importance in the case of MOFs, using probes without strongly interacting functional groups that may compete with the structural coordination bonds minimizes the risk of ligand displacement during measurements.[26–28] Furfuryl alcohol is not expected to break metal–carboxylate bonds.

The well-studied MOF material HKUST-1 is used as an exemplary case. This material is typically obtained by solvothermal treatment of a cupric salt and 1,3,5-benzenetricarboxylic acid in an ethanol–water mixture.[29] When the powder XRD patterns of samples obtained after short and extended crystallization times are compared, both show the diffraction peaks characteristic for HKUST-1 (see Figure S1 in the Supporting Information). In addition, both samples displayed an identical BET specific surface area of (1400±25) m^2^ g^−1^ and no differences were observable based on FTIR spectroscopy or electron microscopy. These observations suggest that for both samples the crystal lattice is largely intact. However, when both samples were evaluated using confocal fluorescence microscopy after incubation with furfuryl alcohol, dramatic differences become apparent. In strong contrast to crystals obtained after a short preparation time (Figure 1 a,b), the crystals that resided in the synthesis mixture for a longer time display intense fluorescence at well-defined zones (see Figure 1 c–f and Movie S1 in the Supporting Information). Note that the very weak and homogeneous fluorescence intensity observed for the intact crystals obtained after a short crystallization time (Figure 1 b) reflects the inactivity of the coordinatively unsaturated copper sites in the HKUST-1 framework for furfuryl alcohol oligomerization. Interestingly, the fluorescent bands and lines in crystals that resided in the synthesis mixture for a longer time seem to be oriented along specific crystallographic directions, as can be seen, for instance, in Figure 1 e. Using scanning probe microscopy, it has been observed that the outer facets of HKUST-1 crystals, which are {111} crystal planes, often display fractures along the 〈110〉 crystallographic directions.[21] Based exclusively on information from surface observations, it has been speculated that such fractures would propagate in the crystal interior either along {111} or {100} crystallographic planes, with the latter being more likely given the smaller number of coordination bonds to be cleaved. Both situations are schematically represented in Figure 2 with indication of the angle the defect plane would make with the {111} crystal facets.

**Figure 1:**
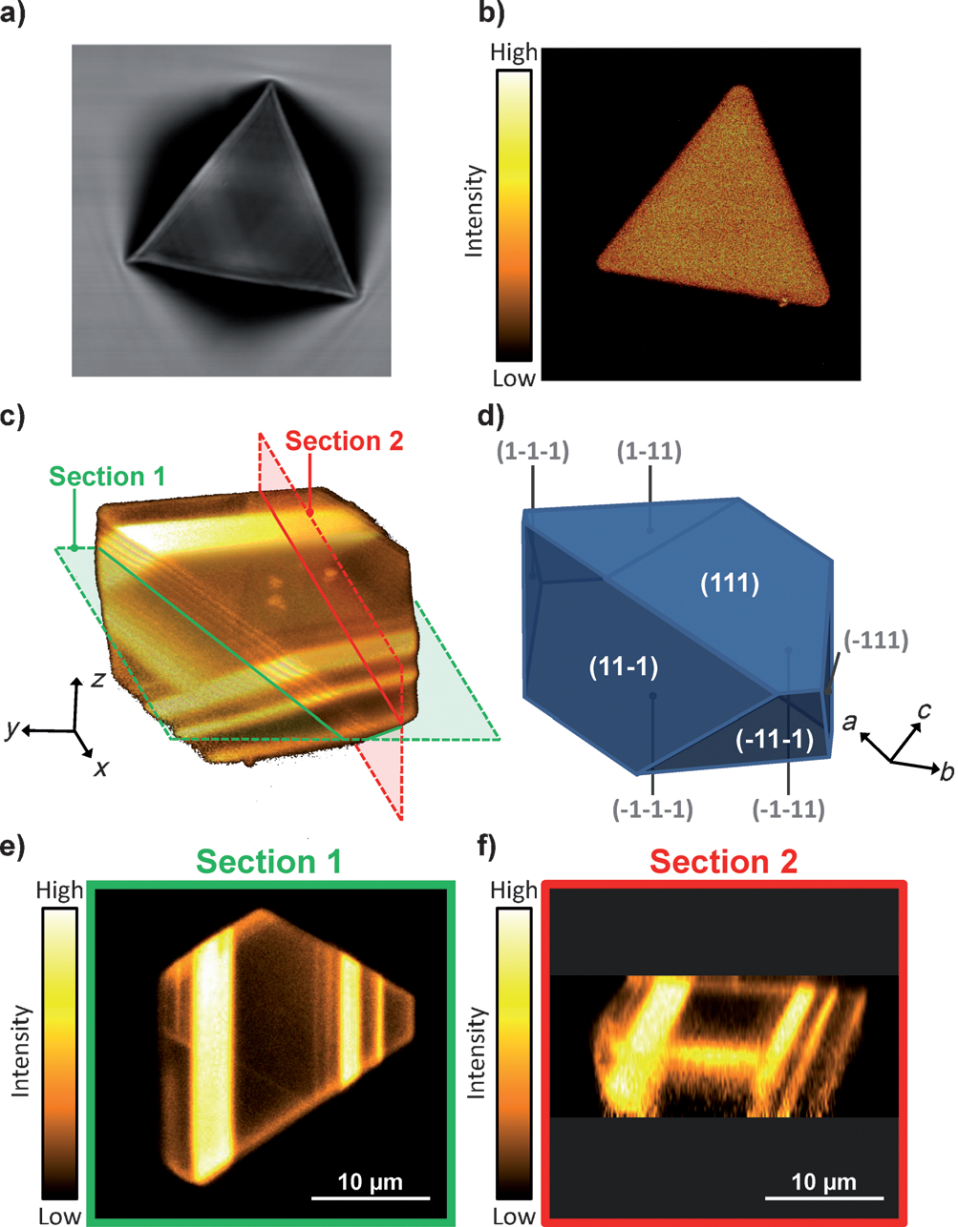
Confocal fluorescence images of HKUST-1 single crystals demonstrating the introduction of defects upon extending crystallization time. a) Transmission micrograph of a HKUST-1 crystal obtained after a short crystallization time. b) Fluorescence micrograph of the surface of the same crystal illustrating the absence of defects. c) Fluorescence data recorded for a HKUST-1 crystal obtained after extended crystallization time. Semitransparent three-dimensional representation of the fluorescence data obtained as a series of *xy*-scans along the *z*-axis. Positions of *xy*- and *xz*-sections shown in panels (e) and (f), respectively, are indicated. d) Schematic representation of the single crystal shown in panel (c) with indication of the limiting crystal planes and crystallographic axes. e) As-recorded *xy*-section. The crystal boundaries in this two-dimensional section are all parallel to the 〈110〉 directions. d) *xz*-section reconstructed from the data shown in panel (c) and visualizing the angles between the crystal’s exterior surface and defect planes propagating in the crystal’s interior.

**Figure 2:**
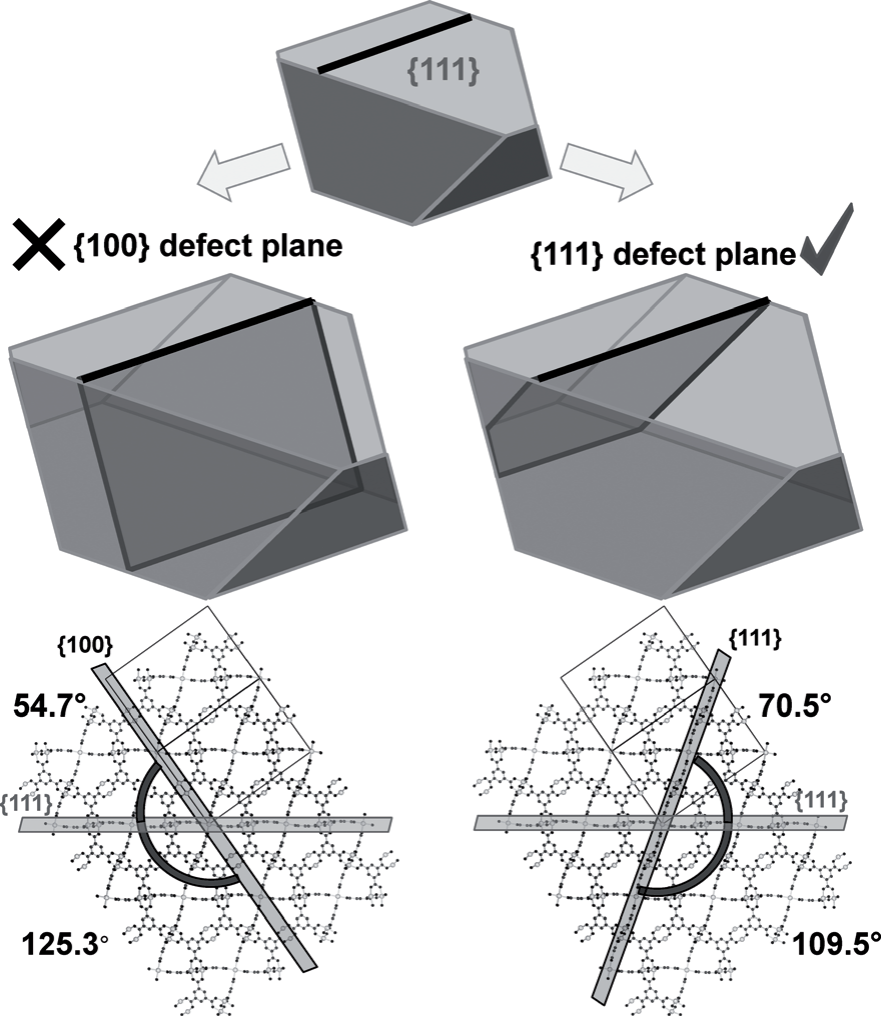
Schematic representation of defect planes that can intersect the outer {111} surfaces of HKUST-1 crystals along <110> directions. Defect planes that belong to the {100} and {111} family of crystallographic planes are indicated in darker gray on the right and the left, respectively. The angles these planes make with {111} planes are indicated in the bottom panel. Confocal fluorescence microscopy data strongly suggests the {111} nature of these defect planes.

The true power of confocal fluorescence microscopy lays in its capability for three-dimensional imaging, which allows visualization of the defects resulting from prolonged synthesis time also in the crystal interior.[30–32] From the resulting data (see Figure 1 and Movie S1 in the Supporting Information) the actual angle between the crystal surface and the defect regions can be readily determined as 70.6°. With the angles indicated in Figure 2 in mind, this result clearly demonstrates that the defect regions in this example are formed along {111} crystallographic planes and not along {100} planes. The latter scenario would result in an angle of 54.7° between the crystal surface and the defect plane, which is evidently not in line with the experimental results. Notably, this result is not what would be intuitively expected based on the number of cleavable coordination bonds in the respective planes.[21] It is likely that other factors such as the accessibility of cleavable bonds play a role as well. Like in previous studies, fluorescence microscopy reveals heterogeneity within the sample.[33–35] Although the vast majority of HKUST-1 crystals display the planar-type defects discussed above after extended crystallization times, some exceptions show other types of defects that are strictly confined to the crystal interior and thus hard to observe using surface imaging techniques (Figure S2).

In order to link the microscopic observations described above to macroscopic properties, a series of bulk catalytic experiments were performed using batches of HKUST-1 obtained after different synthesis times (see Figure 3). Two mechanistically different reactions were studied, both catalyzed or influenced by Brønsted acidity. The first reaction, namely furfuryl alcohol oligomerization, is the same as in the microscopic observation of individual crystals, but now the product formation by a collection of crystallites is quantified by bulk liquid fluorimetry. In accordance with the microscopic observations, the ensemble measurements clearly indicate an increased activity for the conversion of furfuryl alcohol to fluorescent products by HKUST-1 samples obtained after a prolonged crystallization time. The second reaction is the isomerization of α-pinene oxide to campholenic aldehyde, an intermediate in the fragrance industry.[36] This conversion has been shown to be a sensitive probe for the Lewis/Brønsted acid nature of catalysts.[37, 38] While a Lewis acid efficiently catalyzes the formation of campholenic aldehyde, the presence of Brønsted acidic sites lowers the selectivity toward this product. In the case of HKUST-1, samples obtained after a longer synthesis time systematically display a lower selectivity for campholenic aldehyde while more products of Brønsted acid reactions are observed. Summarizing, the trends observed in both bulk catalytic experiments are strong indicators for the increased Brønsted acid defect content in samples subjected to a longer solvothermal treatment. Thus, the microscopic observations of defects at the level of individual crystals were successfully related to the macroscopic catalytic performance of an ensemble of crystals.

**Figure 3:**
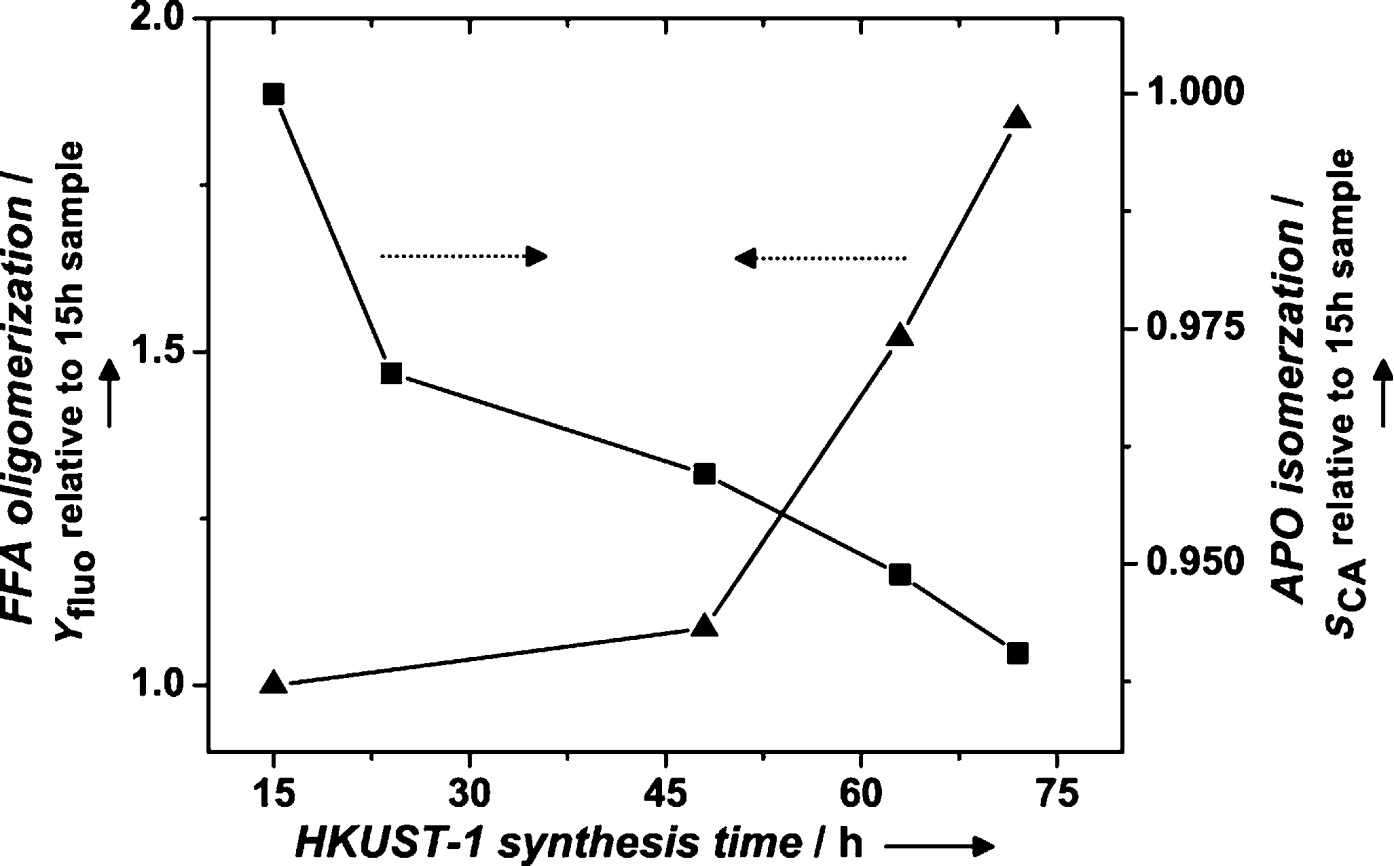
Bulk catalytic experiments demonstrating the increasing Brønsted acid defect content of HKUST-1 samples with increasing synthesis time. For the oligomerization of furfuryl alcohol (FFA) the total yield of fluorescent products (*Y*_fluo_) after 24 h of reaction is plotted (triangles). For the isomerization of α-pinene oxide (APO) the selectivity toward campholenic aldehyde (*S*_CA_) after 2 h of reaction is plotted (squares). Both *Y*_fluo_ and *S*_CA_ are plotted as relative values to the results for the HKUST-1 sample obtained after a short crystallization time of 15 h. The absolute value of *S*_CA_ for that sample is 84.1 %. Both the increase in *Y*_fluo_ and decrease in *S*_CA_ are strong indicators for the increased Brønsted acid defect content in samples subjected to a longer solvothermal treatment.

As an illustration of the general applicability of confocal fluorescence microscopy for studying carboxylate-based MOFs using furfuryl alcohol as a probe, two other materials were studied. As a first example, defects in single crystals of MOF-5, a porous zinc terephthalate, were studied. Fluorescence microscopy reveals the presence of square-shaped defects on the outer surface of MOF-5 crystals (see Figure 4). Although further study is required, such features could be related to the growth mechanism of the MOF-5 crystal faces, as has been previously suggested for other coordination compounds.[39] Importantly, although these square-shaped features are also observed using electron microscopy, Figure 3 d clearly illustrates how drying during sample pretreatment can introduce additional defects. In contrast, fluorescence microscopy can be used to image samples submersed in liquid and thus circumvents crack formation when less robust porous materials are dried. In addition, the three-dimensional capabilities of confocal fluorescence microscopy make it possible to visualize the penetration of defects into the crystal interior (Figure 4 c and Movie S2 in the Supporting Information). In contrast to the defects in the HKUST-1 sample above, the defects in MOF-5 do not propagate throughout the crystal interior but rather seem localized in a 10 μm thick shell.

**Figure 4:**
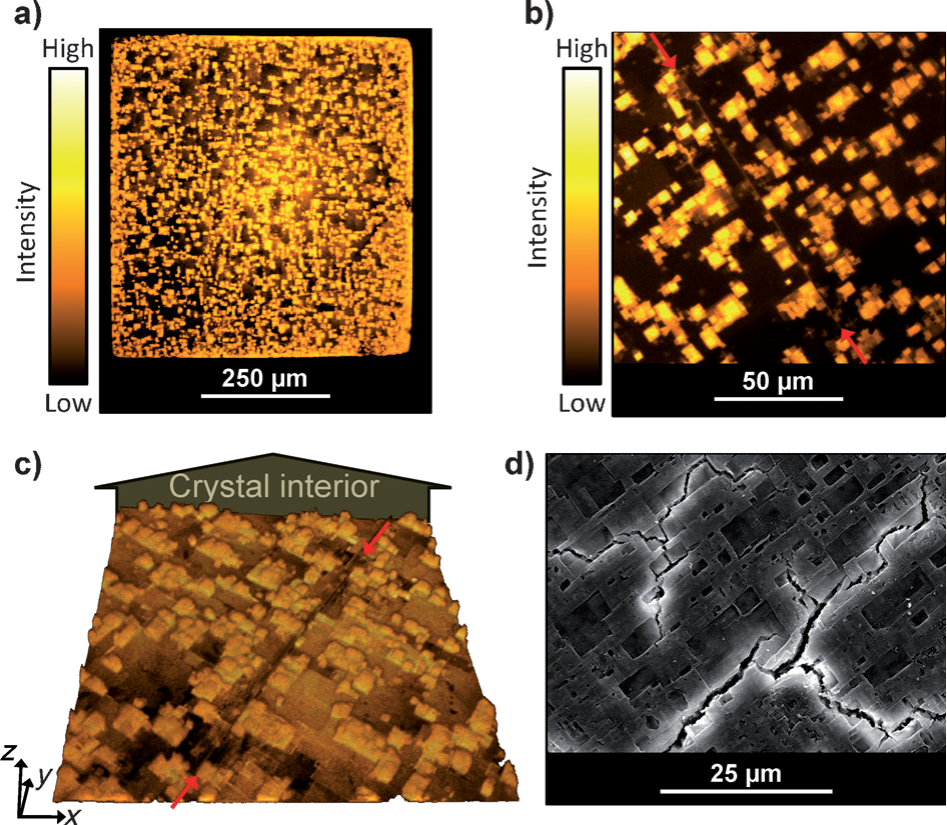
Confocal fluorescence imaging of defects in a MOF-5 single crystal. a) Overview fluorescence micrograph of the outer surface of a large cubic MOF-5 crystal. b) More detailed fluorescence micrograph of the crystal facet shown in panel (a). The red arrows indicate a line defect. c) Three-dimensional representation of the fluorescence data presented in panel (b), illustrating the penetration of defects into the crystal interior. For easy comparison, the same line defect as in panel (b) is indicated with red arrows. Note that in this representation the outer crystal surface is viewed from inside the crystal for optimal visualization of defect penetration. d) Electron micrograph of a similar MOF-5 crystal illustrating the large irregular cracks formed upon drying.

A second example illustrates how defects can be visualized based on reaction with furfuryl alcohol even in materials in which structural Brønsted acid sites are present in the intact framework. MIL-53(Ga) is a member of the MIL-53 family of materials based on gallium and terephthalate linkers.[40] The bridging hydroxyl groups in this material, which are present as a part of one-dimensional chains of corner-sharing GaO_4_(OH)_2_ octahedra, display Brønsted acid character.[41] Nevertheless, since the acidity of these hydroxyl sites is weaker than that of the carboxylic acid defects in the material, their presence does not hinder the visualization of the latter through the acid-catalyzed furfuryl alcohol probe reaction (see Figure S3 in the Supporting Information). It should be noted that the probe reaction used in this work is aimed specifically at visualizing defects in MOFs based on carboxylate linkers. While this certainly covers one of the most significant classes within the pool of MOF materials,[8] it would be interesting to develop similar defect-indicating reactions for materials based on other linker chemistries.

In summary, we have demonstrated how confocal fluorescence microscopy can be a unique tool to study defects in MOF materials. In contrast to currently available techniques, confocal fluorescence microscopy offers the advantage of three-dimensional imaging at the single-crystal level combined with the sensitivity required to study the start of defect formation. Apart from three-dimensional imaging capabilities, the main merit of confocal fluorescence microscopy, as compared to other microspectroscopic techniques probing, for instance, vibrational modes, is its inherent sensitivity when a suitable fluorescent label is employed.[42] In contrast, imaging based on vibrational spectroscopic techniques does not require labeled probes if the spectroscopic signatures of the molecules in the process to be studied are sufficiently different from the absorption bands of the porous host.[31, 43] For in situ experiments simulating, for instance, realistic process conditions, not having to add probe molecules to the reaction mixture is advantageous. However, such techniques might be less suitable to study defects present in low concentrations and buried within the porous framework itself, especially when the vibrational modes of defect sites and framework are similar. In such a case, much more detailed imaging is achieved when the chemical differences of the defects are exploited to selectively highlight them with a fluorescent label rather than aiming at directly visualizing the difference in chemical properties itself.[32] In addition, this study shows how the presence of defects observed at the single-crystal level extrapolates to changes in macroscopic catalytic performance of the whole synthesis batch.

## Experimental Section

HKUST-1 synthesis mixtures were prepared according to literature procedures.[29] The synthesis mixture was loaded in Teflon-lined steel autoclaves and placed in a preheated oven at 383 K for a crystallization time of 15, 24, 48, 63, or 72 h. The samples obtained after 15 and 63 h are indicated as “short” and “extended” crystallization times, respectively, and used for comparison in fluorescence microscopy. Afterwards, samples were cooled to room temperature, separated by filtration, and washed twice with ethanol. After washing, HKUST-1 samples were dried, first at 333 K and subsequently at 393 K, and kept in airtight containers. Nitrogen adsorption on HKUST-1 samples was measured at 77 K using a Quantachrome AS1 instrument. Powder XRD patterns were recorded on a STOE STADI P Combi instrument in Debye–Scherrer geometry (Cu-K_α1_) using a IP-position-sensitive detector (2*θ*=0–60°, Δ2*θ*=0.03°).

Bulk catalytic experiments were performed as follows. For the furfuryl alcohol oligomerization test reaction 2.5 mg of each HKUST-1 sample was incubated in 200 μL of vacuum-distilled furfuryl alcohol at 353 K for 24 h. After 24 h, 10 μL of the supernatant liquid was diluted in 2 mL of spectrograde ethanol and the fluorescence upon excitation at 365 nm was quantified using a Horiba Jobin Yvon Fluorolog fluorimeter. Control reactions using no catalyst, CuO, or Cu_2_O were found to give a significantly lower activity. For the α-pinene oxide test reaction 100 mg of each HKUST-1 sample was incubated with 100 mg of substrate in 5 mL of dichloromethane at 313 K for 2 h. Reaction products were analyzed by gas chromatography using nonane as an internal standard.

MOF-5 samples were prepared according to the procedure reported in reference [44]. After the synthesis solvent was exchanged with chloroform, the crystals were not dried but instead kept under chloroform in airtight containers.

Samples for fluorescence microscopy were prepared by incubating MOF crystals in vacuum-distilled furfuryl alcohol for 18–24 h at 353 K. After this incubation step, crystals and liquid were transferred to a sealed custom measurement cell with glass windows along the beam path. An Olympus Fluoview FV-1000 instrument was used for recording fluorescence micrographs. Fluorescence data were processed using ImageJ software.[45] Crystal models were generated using WinXMorph software.[46]
