# Supplementary material for: Three-Dimensional Visualization of Defects Formed during the Synthesis of Metal–Organic Frameworks: A Fluorescence Microscopy Study
Source: Angew Chem Int Ed Engl. 2012 Nov 9;52(1):401–5. doi: 10.1002/anie.201205627 (PMC4464535; doi:10.1002/anie.201205627)
Supplement: Supplementary file 1 [file anie0052-0401-sd1.pdf]

Angewandte Chemie
Eine Zeitschrift der Gesellschaft Deutscher Chemiker

Supporting Information

© Wiley-VCH 2012

69451 Weinheim, Germany

Three-Dimensional Visualization of Defects Formed during the Synthesis of Metal–Organic Frameworks: A Fluorescence Microscopy Study**

*Rob Ameloot, Frederik Vermoortele, Johan Hofkens, Frans C. De Schryver, Dirk E. De Vos, and Maarten B. J. Roeffaers**

anie_201205627_sm_miscellaneous_information.pdf
anie_201205627_sm_movie_cubtc.avi
anie_201205627_sm_movie_mof-5.avi

Movie S1. Rotating three-dimensional representation of the fluorescence data recorded for the single HKUST-1 crystal shown in Figure 1 in the main text.

Movie S2. Rotating three-dimensional representation of the fluorescence data recorded for the surface of the single MOF-5 crystal shown in Figure 3 in the main text.

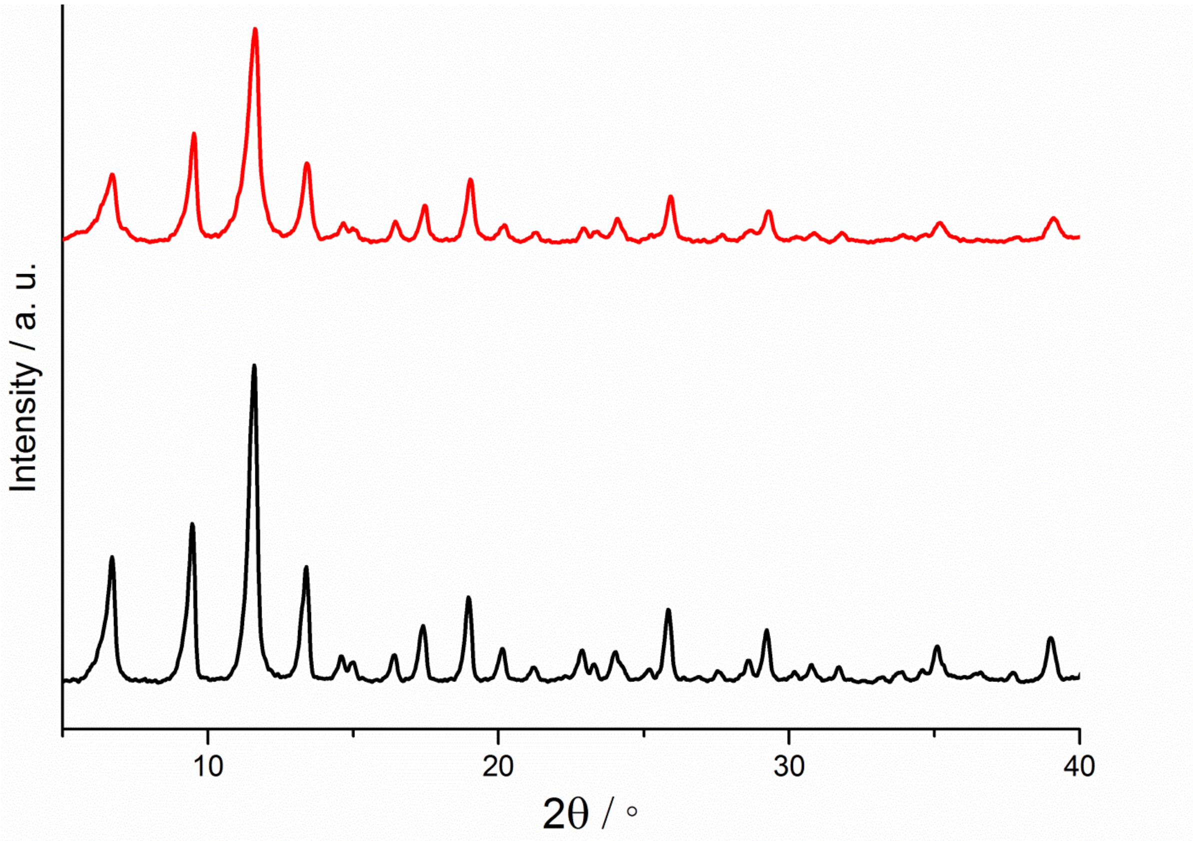

Figure S1. Powder XRD patterns of HKUST-1 samples obtained after a short (red) and extended (black) crystallization time of respectively 15 and 63 hours. Both samples show all diffraction peaks characteristic for HKUST-1.

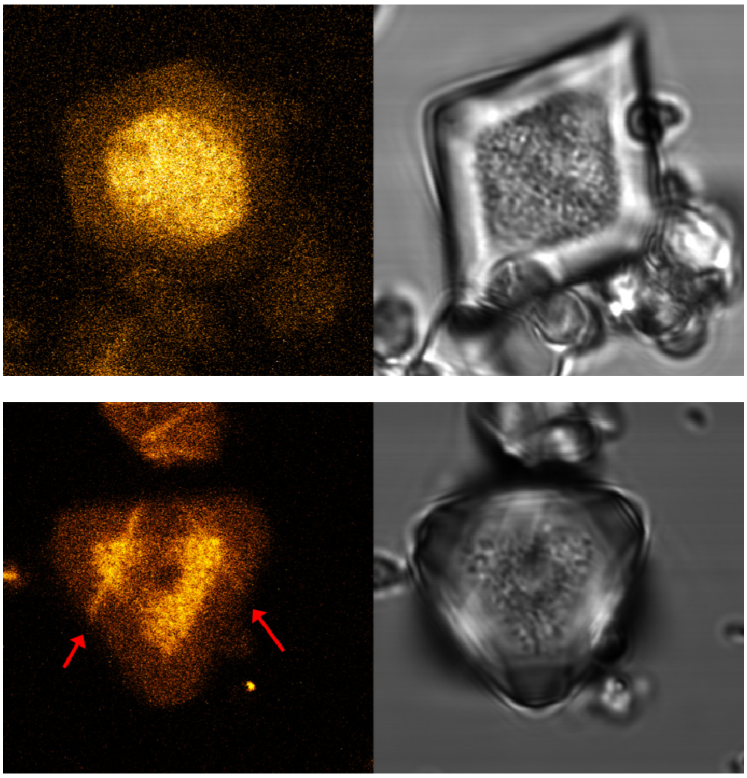

Figure S2. Heterogeneity of defect types observed in some HKUST-1 crystals after extended crystallization. A confocal fluorescence image and an optical micrograph of the same crystal are shown respectively on the left and the right side. The crystal on top shows a high defect density confined in the crystal interior. The crystal on the bottom displays a similar region combined with planar-type defects (red arrows).

Synthesis of MIL-53(Ga)

MIL-53(Ga) was synthesized using a literature procedure[1].
A mixture of $Ga(NO_3).H_2O$ (Pura Tronic, 99,999%), terephthalic acid (H_2BDC, Acros, 99%), HF (Chem-Lab, 40 wt%) and deionized water in a 1:2:1:100 molar ratio was placed in a Teflon-lined stainless steel autoclave. The mixture was heated to 220°C for 3 days. After filtration, the solid was washed twice with hot DMF in order to remove unreacted H_2BDC molecules, once with ethanol and dried at room temperature.

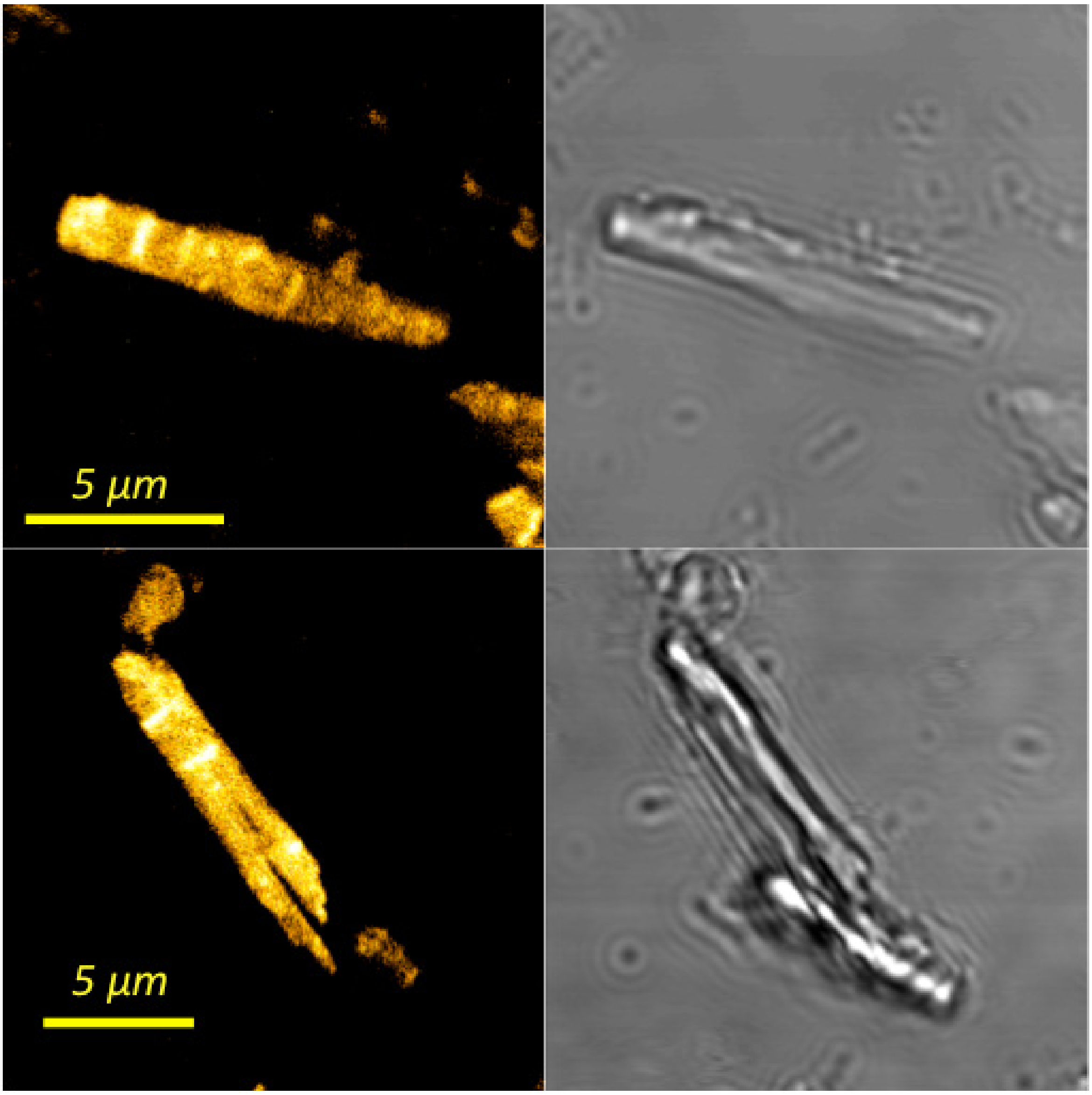

Figure S3. Visualization of defects in crystals of MIL-53(Ga). The structural hydroxyl sites present in the crystal lattice as part of one-dimensional chains of corner-sharing $GaO_4(OH)_2$ octahedra do not hinder the visualization of carboxylic acid defects in the material.

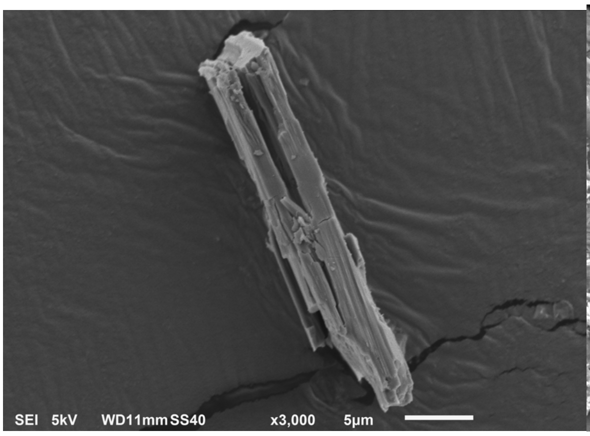

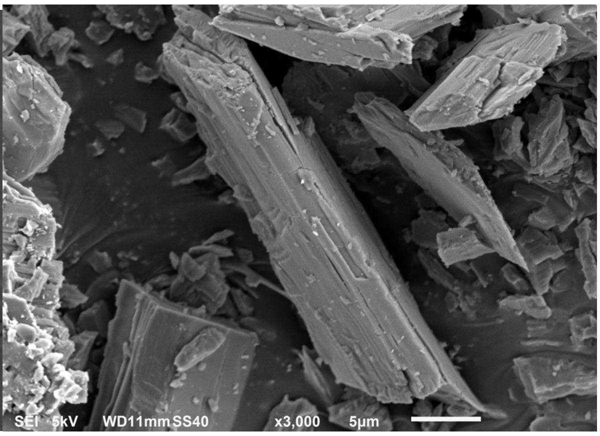

Figure S4. Scanning electron micrograph of individual MIL-53(Ga) crystallites. Large single crystals (left), suitable for confocal microscopy, were selected from the bulk (right).

References

[1] G. Chaplais, A. Simon-Masseron, F. Porcher, C. Lecomte, D. Bazer-Bachi, N. Bats, J. Patarin*, Phys. Chem. Chem. Phys.* **2009**, *11*, 5241–5245
